# Impact of Bimaxillary Advancement Surgery on the Upper Airway and on Obstructive Sleep Apnea Syndrome: a Meta-Analysis

**DOI:** 10.1038/s41598-018-24142-3

**Published:** 2018-04-10

**Authors:** Carolina Rojo-Sanchis, José Manuel Almerich-Silla, Vanessa Paredes-Gallardo, José María Montiel-Company, Carlos Bellot-Arcís

**Affiliations:** Department of Stomatology, Faculty of Medicine and Dentistry, University of Valencia, Valencia, Spain

## Abstract

Upper airway changes following bimaxillary advancement surgery to treat obstructive sleep apnea syndrome remain controversial. The main objective of this systematic review and meta-analysis was to investigate the effects of bimaxillary advancement surgery on the upper airway (UA) of obstructive sleep apnea syndrome patients through examining changes three-dimensionally in vertical and supine position and through changes in oximetric variables (AHI, RDI, O2 Sat) and in the quality of life measured by the Epworth sleepiness scale (ESS). A thorough search of the PubMed, Scopus, Embase and Cochrane databases and a grey literature search (Opengrey) were conducted. No limit was placed on publication year or language. The inclusion criteria were: adult obstructive sleep apnea patients who had undergone bimaxillary advancement surgery, three-dimensional CBCT or CT and oximetric measurements and at least six weeks follow-up. Sample sizes of under 10 patients were excluded. Finally, 26 articles were included in the qualitative review and 23 in the meta-analysis. Bimaxillary advancement surgery has been shown to be beneficial in terms of increased upper airway size, improved oximetric indicators and the quality of life measured on the Epworth sleepiness scale.

## Introduction

The incidence of obstructive sleep apnea syndrome (OSA) is 5–25% in adults^1^, or 2–4% in men and 1–2% in women^2^. This condition has serious consequences in terms of cardiovascular and metabolic function, lower quality of life and neurocognitive impairment^3^. The complications may be related to lower saturation of hemoglobin levels during sleep. A smaller upper airway (UA) volume or the presence of constrictions that present greater resistance to air passing through it are considered risk factors for OSA development^4^, owing to the occurrence of oxygen excitation or desaturation^1,5,6^.

The physiopathology of OSA has been related to predisposing anatomical factors such as craniofacial anomalies, macroglossia, hypotonia of the soft tissues of the oropharynx, retroposition of the base of the tongue, mandibular hypoplasia and retroposition and maxillary retrusion^7^. Additionally, pharyngeal obstruction is commonly found in patients with retrognathia or a dolichofacial appearance^8^.

OSA is classified by the apnea/hypopnea index (AHI). It is considered mild when the number of events per hour is between 5 and 20, moderate with 20 to 35 events per hour and severe when the apnea/hypopnea index is over 35. An AHI of 5 or under is considered normal in an adult^5^.

Maxillofacial operations on patients with a severe form of OSA are increasing owing to the high success rate of maxillomandibular advancement^9–12^, even in very severe cases with AHI scores of over 100^13^. The literature contains solid evidence of improvement following maxillomandibular advancement^1,4,7,14–17^. Nevertheless, standardized anatomical limits and methods to determine the area need to be established before the real benefits of this type of surgery can be clarified^4^. Additionally, the position of the patient when the airway is measured after surgery appears to be decisive, and this aspect has not been taken into account in previous studies^4,18^.

## Objectives

The main objective of this systematic review and meta-analysis was to investigate the effects of bimaxillary advancement surgery on the UA of patients with obstructive sleep apnea syndrome. This was effected through three-dimensional examination in vertical and in supine position and changes in oximetric variables (AHI, RDI, O2 Sat) and in the patient’s quality of life, measured on the Epworth sleepiness scale (ESS).

## Materials and Methods

The systematic review was conducted in accordance with the PRISMA guidelines (Preferred Reporting Items for Systematic Reviews and Meta-Analyses)^19^ and was previously registered with PROSPERO under registration number CRD42017064891.

### PICO question

The objective was to answer the following research question: What effects does bimaxillary surgery have on the upper airway space in patients with obstructive sleep apnea, on comparing the pre- and postoperative dimensions?

### Inclusion and exclusion criteria

“Articles” and “articles in press” were included. Randomized clinical trials (RCTs), cohort studies and case -controlled studies were included. Retrospective and prospective studies were included. No restriction was placed on publication year or language. The inclusion criteria were: studies of adult patients with obstructive sleep apnea who had undergone bimaxillary advancement surgery, with three-dimensional CBCT or CT and oximetric measurement records and a follow-up period of at least six weeks. Studies with sample sizes of fewer than 10 patients were excluded.

### Search Strategy

#### Sources of information

To identify the potentially relevant studies irrespective of language, a thorough electronic search was made in the PubMed, Scopus, Embase, and Cochrane databases. An electronic search of grey literature was made through Opengrey. In particular cases the authors of the articles were contacted by email to request missing information. The reference lists of the studies included were hand-searched to identify and examine articles not found in the databases that might meet the inclusion criteria. This systematic review and meta-analysis was updated in March 2017.

#### Search terms

The search strategy included 12 Mesh (Medical Subject Heading) terms: “Malocclusion, Angle Class II”, “Orthognathic surgery”, “Mandibular advancement”, “Airway”, “Upper Airway”, “PAS”, “Nasopharynx”, “Oropharynx”, “Hypopharynx”, “Hyoid bone”, “Obstructive sleep apnea”, “OSA” and 4 uncontrolled descriptors: “Posterior airway space”, “Pharyngeal space/airway”, “Retrognathia”, and “Bimaxillary surgery”. Boolean operators (“OR” and “AND”) were used to join terms (MeSH/non-MeSH) related to the research question.

These keywords were divided into two groups: 5 primary keywords related to orthognathic surgery terminology and 11 secondary keywords related to UA and OSA. Searches were made for all the possible combinations between the terms in the two groups, separately and combined (Appendix Table 1). The articles identified were exported to Mendeley Desktop 1.13.3 software (Mendeley Ltd, London, England) to check for duplicates.

#### Study selection

Two reviewers (CR-S and CB-A), working independently, systematically assessed the titles and abstracts of all the articles identified If they disagreed, a third reviewer was consulted. If the abstract did not contain sufficient information to reach a decision, the reviewers read the full article before taking the final decision. Subsequently the full texts of all the articles were read and the reasons for rejecting those excluded were recorded (Appendix Table 2).

#### Study data

The following variables were recorded for each article: author and year of publication, type of study, sample size (including losses to follow-up) and demographic variables (gender and age). To classify the severity of the OSA, the pre-and post-operative body mass index (BMI), apnea/hypopnea index (AHI), oxygen saturation (O2 Sat), respiratory disturbance index (RDI) and Epworth sleepiness scale (ESS) scores were recorded.

Any previous surgery on the patient was also recorded, as was the type of operation performed and any additional operation carried out during the process, together with the maxillomandibular advancement length and upper airway changes at the follow-up examinations. The method used to study pre-and postoperative changes was classified according to the radiographic method (two-dimensional or three-dimensional) and whether a polysomnograph was used.

### Quality assessment

The quality of the studies was assessed by the same researchers, working independently, using the Newcastle-Ottawa Scale^20^. Any discrepancy between the initial two researchers was settled by consensus and where doubts remained the third researcher was consulted.

### Measurement of the variables and synthesis of the results

The initial and final means and confidence intervals were recorded for the following variables: UA in vertical position, UA in recumbent position, AHI, RDI, O2 Sat and ESS.

### Statistical analysis

For the quantitative synthesis, the differences between the initial and final means were calculated, together with their confidence intervals. Heterogeneity was assessed by the Q test and the I^2^ statistic. A Q test p-value of less than 0.1 was considered heterogeneous. In that case, the random effects method was used to calculate the difference in means. Publication bias was measured by funnel plots and the classic fail-safe number. The software employed was comprehensive meta-analysis V 3.0 Biostat.

## Results

### Study selection and flow diagram

The search identified 2979 preliminary references related to changes in the airway following orthognathic surgery, of which 1410 were found in Pubmed, 640 in Scopus, 4 in Cochrane, 908 in Embase, 13 in the grey literature search and 4 through hand-searching based on the references cited in the articles included. After excluding 2629 duplicates, the remaining 350 were screened. Of these, 297 were excluded on reading the title and abstract as they were unrelated to the research question. After examining the full text of the resulting 53 articles, 27 were excluded for the following reasons: 13 did not answer the PICO question, 7 only examined the UA two-dimensionally, 5 were narrative reviews or letters to the editor, 1 included patients aged under 18 years and 1 had a sample size of fewer than 10 patients. Finally, 26 articles met the inclusion criteria and were included in the qualitative review, and 23 were included in the quantitative review (meta-analysis). The PRISMA flow chart (Appendix Fig. 1) gives an overview of the article selection process.

### Characteristics of the studies included

The studies included in the systematic review examined a minimum of 10 patients. The largest ones were Boyd *et al*.^17^ who divided their sample into two subgroups, one of 37 patients who underwent maxillomandibular advancement surgery (MMA) and another of 35 patients who underwent both MMA and uvulopalatopharyngoplasty, and Riley *et al*.^21^ whose 40 patients underwent bimaxillary advancement surgery. Not all the papers mentioned whether the sample included patients who snored, smoked, and/or drank alcohol, but most reported the mean age, gender and body mass index. In the studies included in the present review the patients were all adults, with a mean age of approximately 45 years; only Faria *et al*.^7^ and Hernández-Alfaro *et al*.^22^ did not report this variable.

Out of the 26 studies (7 prospective and 19 retrospective), 25 were cohort studies and one was a case-control study.

Most of the articles presented medium-high quality on the Newcastle-Ottawa scale(Appendix Table 3)^20^. Of the cohort studies, four scored 5/9, indicating medium quality^15,22–24^ and eight scored 6/9^8,10,17,21,25–28^. A higher score, 7/9, was achieved by nine studies^7,13,29–35^. The highest-scoring cohort studies were Ronchi *et al*.^36^, Faria *et al*.^11^, Bianchi *et al*.^16^ and Zinser *et al*.^37^ with 8/9. However, Butterfield *et al*.^38^, the only case-control study, achieved the maximum possible score (9/9).

### Qualitative synthesis of the studies included

The mean advancement effected by the surgery was between 4.1 and 10 mm in the maxilla and between 6 and 12.9 mm in the mandible.

The upper airway was a parameter included in all the studies. Postoperative changes were studied through cephalometry alone in 9 of the papers^7,8,10,15,21,24–27^. These changes were also examined three-dimensionally in 13 studies: 7 used CT^16,29–31,33,36,37^, 5 assessed the UA by means of CBCT^22,32,34,35,38^ and 1 did so through MR^11^. Two of the articles measured the changes two-dimensionally, through teleradiography, but also in three dimensions, for which Butterfield *et al*.^34,38^ used CBCT while Ronchi *et al*.^36^ used CT.

The follow-up data from each study were analyzed to assess the surgical process over time, distinguishing four stages: T0 (preoperative), T1 (postoperative), T2 (1^st^ checkup), and T3 (2^nd^ checkup). All the articles assessed the patients prior to surgery, without defining a specific time interval except for Hernández-Alfaro *et al*.^22^, who stated that the preoperative scans were performed one day before surgery. Most of the studies defined the time interval to T2, examining the patients during the first year after surgery. Only 2 articles did not assess that patients at T2^17,32^ but did so at T1, as did Ronchi *et al*.^36^, Riley *et al*.^21^, and Giarda *et al*.^26^. Only Hsieh *et al*.^33^, Riley *et al*.^21^ and Conradt *et al*.^8^ also performed a T3 assessment one or two years after surgery.

Appendix Table 4 presents the studies included, showing type of study, sample size, dropouts, demographic variables, oximetric variables (AHI, RDI, O2 Sat), Epworth scale (ESS), previous surgery (previous ops), changes in upper airway (UA), amount of maxillomandibular advancement (MMA), additional surgery (additional ops), and follow-up time.

### Quantitative synthesis of the studies included

#### Changes in UA dimensions

**Figure 1:**
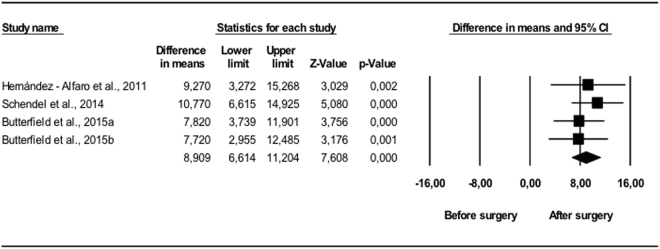
Changes in UA, vertical position (mm^3^). Enlargement of upper airway following bimaxillary advancement surgery. Meta-analysis.

**Figure 2:**
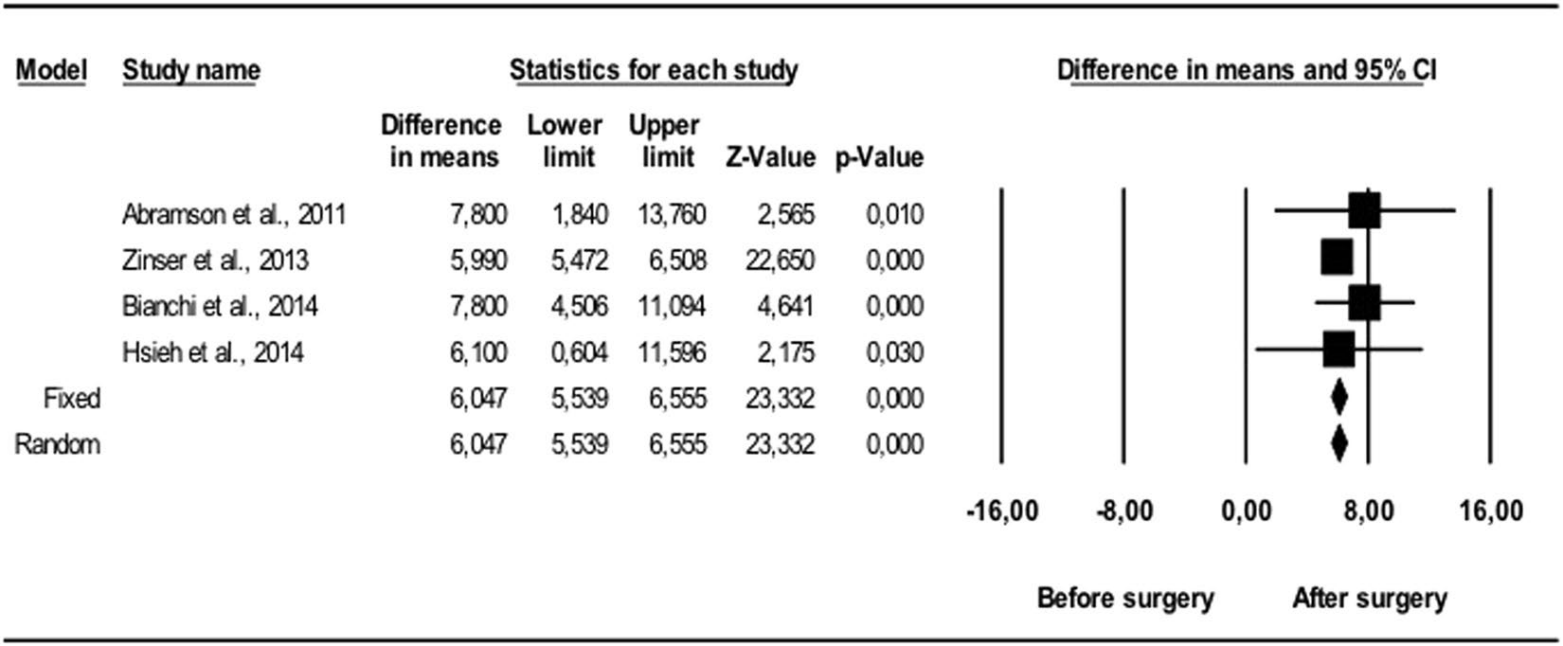
Changes in UA, supine position (mm^3^). Enlargement of upper airway following bimaxillary advancement surgery. Meta-analysis.

Comparing the pre-and postoperative results, changes occurred in all the variables. The UA volume in vertical position (Fig. 1) showed statistically significant (p < 0.01) mean volume increases of between 7.7 and 10.7 cm^3^. The random effects model estimated 8.91 cm^3^, with a 95% CI between 6.61 and 11.2 cm^3^, which was statistically significant (p < 0.001). The I^2^ null value indicated an absence of heterogeneity (Q = 1.29; p = 0.730). The variations measured in supine position (Fig. 2) also showed mean volume increases of between 5.9 and 7.8 cm^3^, all of which were statistically significant (p < 0.05). The random effects model concluded that the difference in means was 6.05 cm^3^ with a 95% CI of 5.54 to 6.56, which was also considered statistically significant (p < 0.001). It was again found that all the studies showed very consistent results (I^2^ = 0), and consequently lacked heterogeneity (Q = 1.47; p = 0.690).

#### Oximetric changes

**Figure 3:**
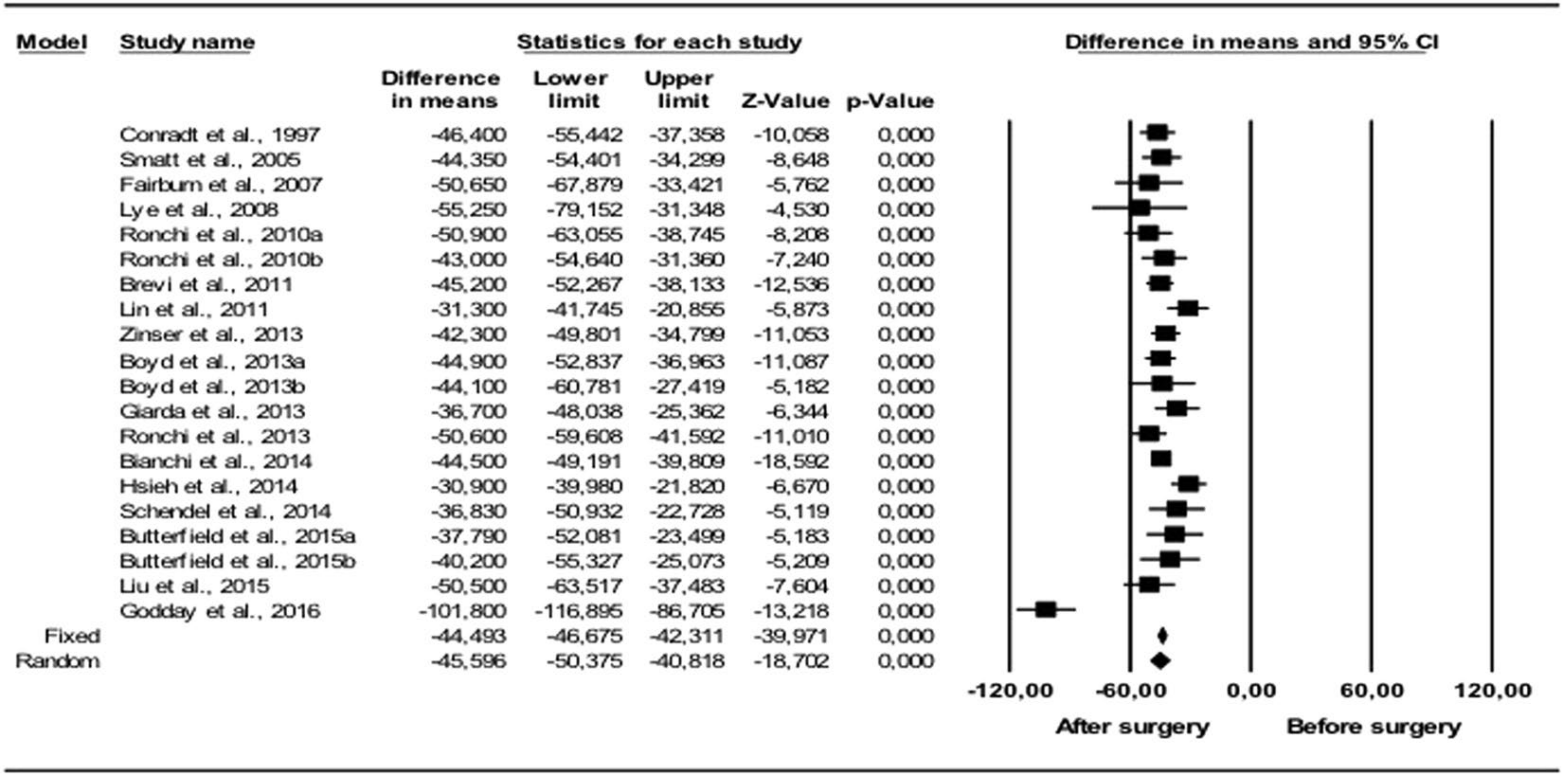
Changes in AHI (events/hour). Decrease in apnea/hypopnea index following bimaxillary advancement surgery. Meta-analysis.

**Figure 4:**
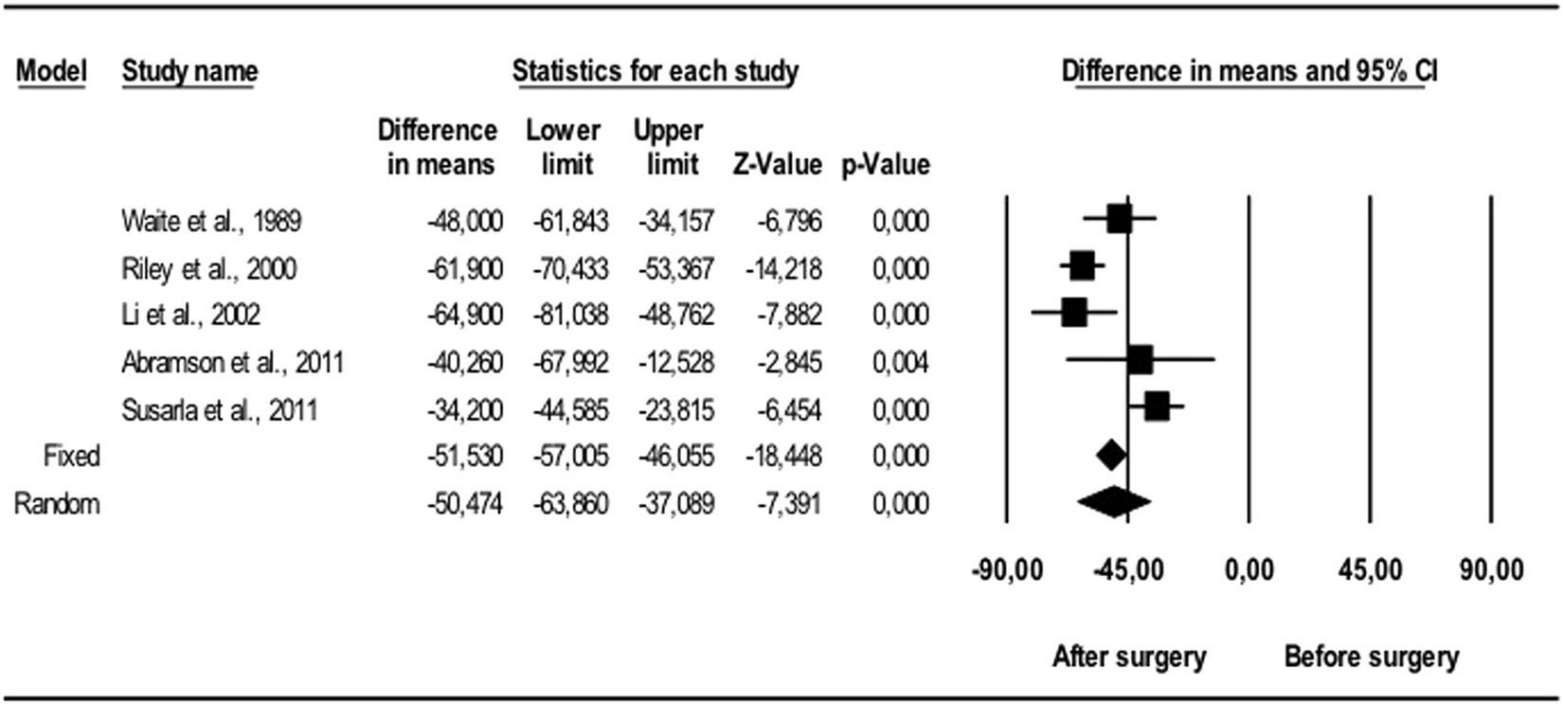
Changes in RDI (events/hour). Decrease in respiratory disturbance index following bimaxillary advancement surgery. Meta-analysis.

**Figure 5:**
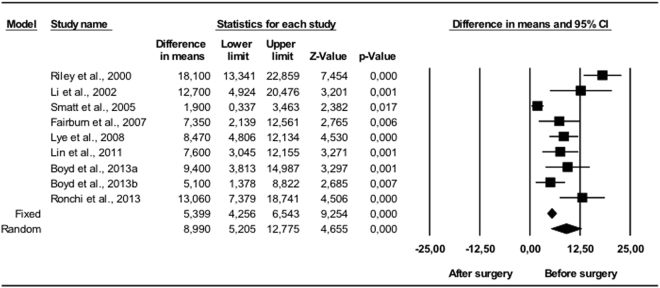
Changes in O2 Sat (%). Increase in oxygen saturation index following bimaxillary advancement surgery. Meta-analysis.

The mean fall in AHI (Fig. 3) was statistically significant (p < 0.001): over 30 events/hour, with a 95% CI of 50.4 to 40.8. The reduction in RDI (Fig. 4) was significant (p < 0.001) in all the studies, with mean values between 34.2 and 64.9 events/hour. The random effects model gave a difference in means of 50.4 events/hour with a 95% CI of 63.9 to 37.1, which again was statistically significant (p < 0.001). Oxygen saturation (Fig. 5) increased following surgery (5.20–12.77); the random effects model estimated a statistically significant (p < 0.001) difference in means of 8.99%, with a 95% CI of 5.21 to 12.8. Heterogeneity was high (I^2^ > 75%) for AHI, RDI and O2 Sat, with I^2^ = 76.2%, I^2^ = 79.9% and I^2^ = 87.3% respectively.

#### Changes in quality of life

**Figure 6:**
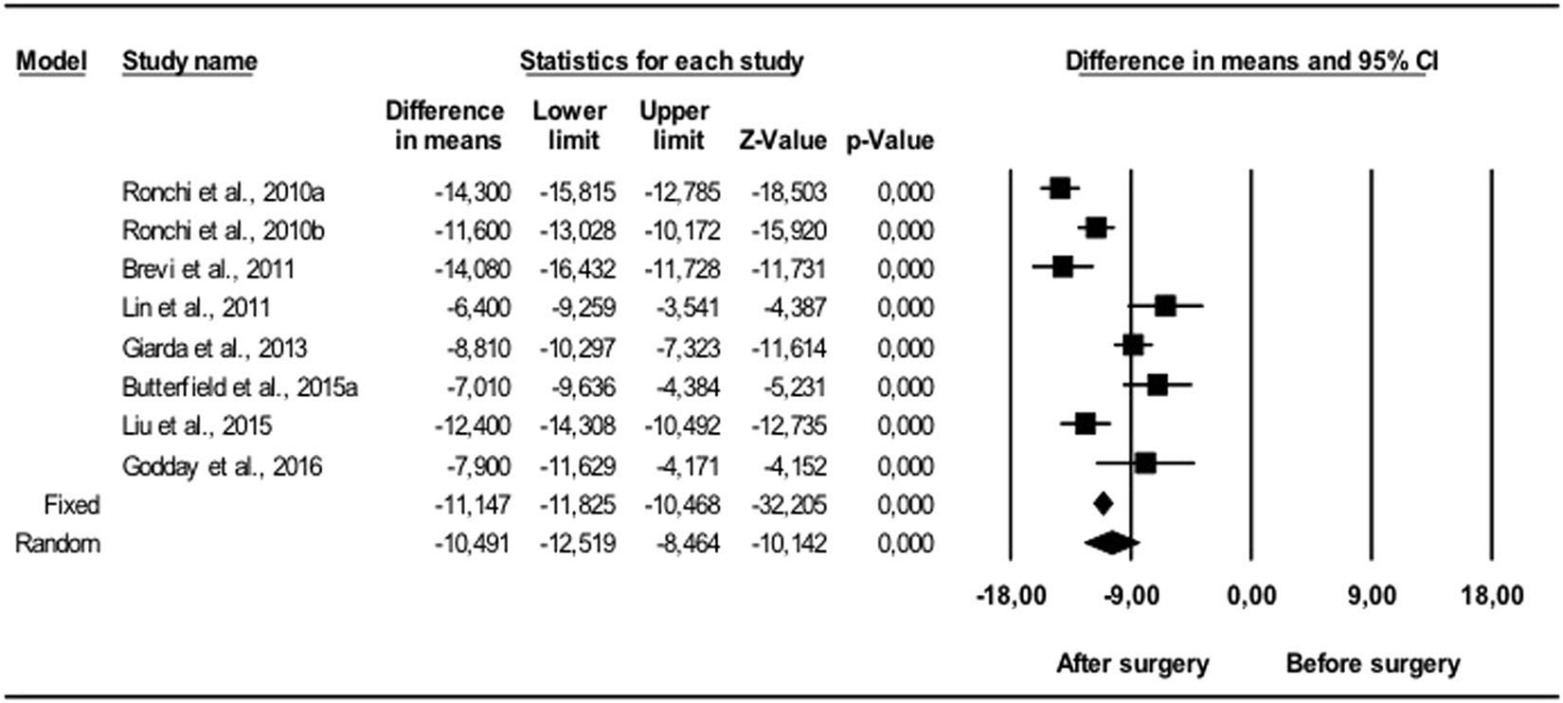
Changes in ESS. Decrease in Epworth sleepiness scale following bimaxillary advancement surgery. Meta-analysis.

In all cases, the Epworth questionnaire (Fig. 6) showed a significant reduction in ESS (p < 0.001). The random effects model concluded that the difference in means was −10.5 (95% CI −12.5 to −8.47), which was statistically significant (p < 0.001). Of all the variables, ESS showed the highest heterogeneity: I^2^ = 87.7% (Q = 57.1; p < 0.001).

### Publication bias

In general, possible publication bias problems were not detected; the classic fail-safe values were quite high for all the variables studied.

As regards increased UA size following surgery, the considerable homogeneity in both vertical and supine position has already been mentioned; it may be seen in the funnel plots for each of these two variables (Appendix Figs 2 and 3) that the four studies in each of the meta-analyses concentrate around the global mean in a totally symmetrical arrangement. There was no publication bias. The number of classic fail-safe studies was estimated at 55 for vertical position and 264 for supine position. This suggests that a large number of articles would have to have not been published for these meta-analyses not to be significant.

The same holds true for the oximetric variables AHI, RDI, and O2 Sat, which would need 7426, 375 and 294 studies respectively to counteract the meta-analysis results (Appendix Figs 4, 5 and 6).

In view of the funnel plot for ESS (Appendix Figure 7), no suspicion of publication bias may be entertained. This was corroborated by a classic fail-safe number of 1840.

## Discussion

Traditionally, obstructive sleep apnea (OSA) is treated by continuous positive airway pressure (CPAP) or mandibular advancement devices (MAD)^32,35^, which do not solve the problem definitively and require patient compliance. These methods are poorly tolerated tending to relapse^26,35,38^. In recent years, other alternatives such as orthognathic surgery have gained greater prominence, so that its evidence is solid in the literature^4,7,14–17^. Moreover, the importance of the surgical treatment of OSA lies in expanding the velopharyngeal way.

As regards changes in upper airway dimensions, the results of the present review show that they vary according to the patient’s posture. The estimated increase in airway space was 8.91 cm^3^ in vertical position but 6.05 cm^3^ in supine position.

Physiologically this is crucial, so it was decided only to study the post-MMA upper airway volume three-dimensionally on the axial plane, perpendicular to the UA; this cannot be viewed by teleradiography^39^. Nowadays, CBCT affords high precision, easy handling and lower radiation doses, and is widely used to examine the UA^4^.

When assessing the increase in UA size, one important factor is the patient’s position (sitting or lying down) when the image is obtained^22,33,35,40^. It was therefore decided to study the three-dimensional UA data separately for these two positions. The studies that used CBCT to measure the UA^22,34,35,38^ (Appendix Table 5) presented a sitting patient without mentioning respiration control, with the exception of Hernández-Alfaro *et al*.^22^, who had the patient sitting vertically and breathing peacefully with the Frankfort plane horizontal, parallel to the ground, the tongue in a relaxed position and the mandible in centric occlusion, biting on a wax bite wafer to stabilize this relation.

A lack of agreement was also observed regarding standardized head positioning methods during scanning to obtain tomographic images^5,18^. Many authors^16,31,34,35,38^ advocate a natural head position (NHP) and the assistance of a mirror or a laser light during scanning. However, Hernández-Alfaro *et al*.^22^ and Hsieh *et al*.^33^ took the Frankfort plane as their reference. In addition, Zinser *et al*.^37^ asked the patients to stay still during the scan, not to swallow, to place the tongue against the incisors and to hold their breath at the end of exhalation, keeping the mandible positioned centrally and the lips relaxed.

Studies that used CT imaging (Appendix Table 6) controlled the respiration at the end of exhalation^31,33,37^ or mentioned holding the breath at the end of normal inhalation^16^.Centric occlusion with the Frankfort plane perpendicular to the ground, without swallowing and with the mouth closed at the end of exhalation, was chosen by Hsieh *et al*.^33^ as a reproducible head position. Faria *et al*.^11^ was the only study included that used MR to assess the UA with the patient awake. However, it was excluded from the quantitative synthesis to eliminate the possible risk of bias for changes in UA volume, as the scanning time was longer so the breathing control would not be equivalent to that of the other studies.

The time taken to obtain the tomographic images varied between 7 seconds and under 15 seconds, respectively in Hernández-Alfaro *et al*.^22^ and Abramson *et al*.^31^. The other studies included gave no indication of this value^11,16,31,34,35,37,38^. Not all the studies reported results in the same units: some expressed the increase in UA diameter in cubic centimeters or millimeters, others in milliliters and others as a percentage. Furthermore, the studies included did not all use the same anatomical limits to define the airway, for instance, Schendel *et al*.^35^ chose the hyoid as the lower limit while Butterfield *et al*.^34,38^ and Ronchi *et al*.^36^, took the tip of the epiglottis as their reference point for this limit (Appendix Tables 5 and 6).

In the three oximetric variables (AHI, RDI, and O2 Sat), a statistically significant (p < 0.001) improvement following surgery was observed: apnea events/hour during sleep (AHI) fell by 45.6 events/hour, the respiratory disturbance index fell by 50.4 events/hour, and oxygen saturation (O2 Sat) increased by 8.99%. For AHI, the study by Godday *et al*.^13^ was a source of considerable heterogeneity in the meta-analysis, which ceased to be heterogeneous when this study was excluded although the resulting estimate continued to be very similar to that obtained when it had been included. For this reason it was decided to continue to include it, as it met the criteria despite being the only study that included patients with extreme clinical manifestations and obesity. The results of the present review agree with those obtained by Zaghi *et al*.^41^, who concluded that there was a significant improvement in this indicator; unlike the present review, theirs included studies with very small sample sizes, with fewer than 10 patients. The heterogeneity in the respiratory disturbance and oxygen saturation (RDI, O2 Sat) results does not seem to be caused by any one article but to respond to a general overall variability.

The improvement in the patients’ quality of life was studied through the Epworth sleepiness scale (ESS). This comprises a series of questions asking the patient about the possibility of falling asleep in certain situation. A statistically significant (p < 0.001) decrease in this likelihood was estimated. Again, the source of heterogeneity in the Epworth sleepiness scale could not be attributed to any single article. It should be mentioned that the two oldest studies^10,36^ were among the four that reported the greatest improvements in the Epworth scale.

Because many patients with OSA have retrognathia (Class II malocclusion), the mandible is usually advanced more than the maxilla. During maxillomandibular advancement surgery, the usual practice is to advance the maxilla to the maximum point first, then advance the mandible into occlusion^1^. Together with advancement surgery, various authors^5,10,32,34,37^ studied a variation on this process: maxillary rotation, known as counterclockwise rotation (CCW).

A significant lack of agreement in follow-up times after surgery was found^4,18^. They varied between 1 and 12 months, although the most common time for the first post-operative checkup was 6 months. In 1997, Conradt *et al*.^8^ were already pointing to the importance of checking the stability of the initial postoperative results over a specific length of time (2 years). Nevertheless, only 6^8,21,27,33,34,36^ followed up for 1 year or more following bimaxillary surgery. In the case of Conradt *et al*.^8^, a considerable decrease in AHI values was found at T2, 6–12 weeks after surgery (5 ± 5.8 compared to 51.4 ± 16.9 at T0), but they increased slightly in time (8.5 ± 9.4 at T3 = 1–2 years). Riley *et al*.^21^ found higher oxygen saturation 6 months after surgery (85.6 ± 4.1%) than pre-operatively (67.5 ± 14.8), though it fell (80.6 ± 3.9%) at T3, 50.7 ± 31.9 months after surgery. However, the RDI values in the same study decreased progressively over the same time intervals (T0 = 71.2 ± 27.0; T2 = 9.3 ± 5.4; T3 = 7.6 ± 5.1).

One of the main limitations of the present review is the disparity in sample size distribution by gender, age ranges and sleep apnea indices, as this limits the precision of the three-dimensional analysis of the upper airway to the post-operative changes. The scarcity of post-operative results for some variables, a greater number of retrospective studies, and difficulty in collecting data owing to the lack of clear values and the use of different units of measurement, all hindered the present investigation. The absence of consensus on the anatomical reference points, together with variations in breathing control and head position in the different studies, limited the precision in measuring the increased airway volume following bimaxillary advancement. Nevertheless, in all the studies included in the present meta-analysis, three-dimensional assessment of the UA showed a statistically significant increase (p < 0.001) in the upper airway space following surgery.

## Conclusions

These findings confirm the benefits of bimaxillary advancement in terms of the increased total volume of the upper airway, improved oximetric indicators and better quality of life on the Epworth sleepiness scale.

## Electronic supplementary material

Supplementary Information
