# Supplementary material for: Impact of Bimaxillary Advancement Surgery on the Upper Airway and on Obstructive Sleep Apnea Syndrome: a Meta-Analysis
Source: Sci Rep. 2018 Apr 10;8:5756. doi: 10.1038/s41598-018-24142-3 (PMC5893577; doi:10.1038/s41598-018-24142-3)
Supplement: Supplementary file 1 — Supplementary Information [file 41598_2018_24142_MOESM1_ESM.pdf]

IMPACT OF BIMAXILLARY ADVANCEMENT SURGERY ON THE UPPER AIRWAY AND ON OBSTRUCTIVE SLEEP APNEA SYNDROME: A META-ANALYSIS

Carolina Rojo-Sanchis[1], José Manuel Almerich-Silla[1], Vanessa Paredes-Gallardo[1], José María Montiel-Company*[1] & Carlos Bellot-Arcís[1]

APPENDIX FIGURE 1. PRISMA 2009 Flow Diagram

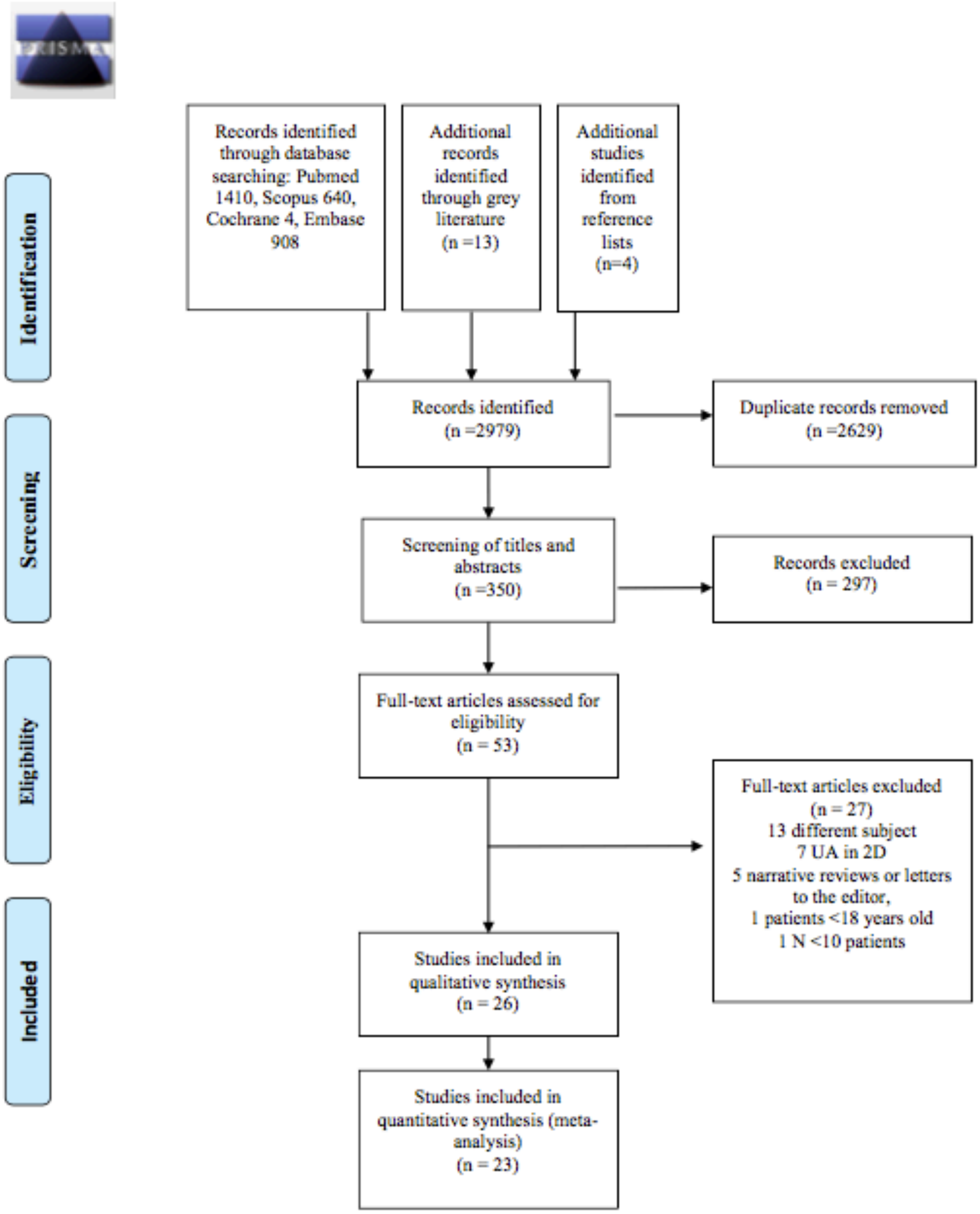

From: Moher D, Liberati A, Tetzlaff J, Altman DG, The PRISMA Group (2009). *Preferred Reporting Items for Systematic Reviews and Meta-Analyses*: The PRISMA Statement. PLoS Med 6(7): e1000097. doi:10.1371/journal.pmed1000097

For more information, visit www.prisma-statement.org.

APPENDIX FIGURE 2. Changes in UA, vertical position (mm^3). Enlargement of upper airway following bimaxillary advancement surgery. Funnel plot

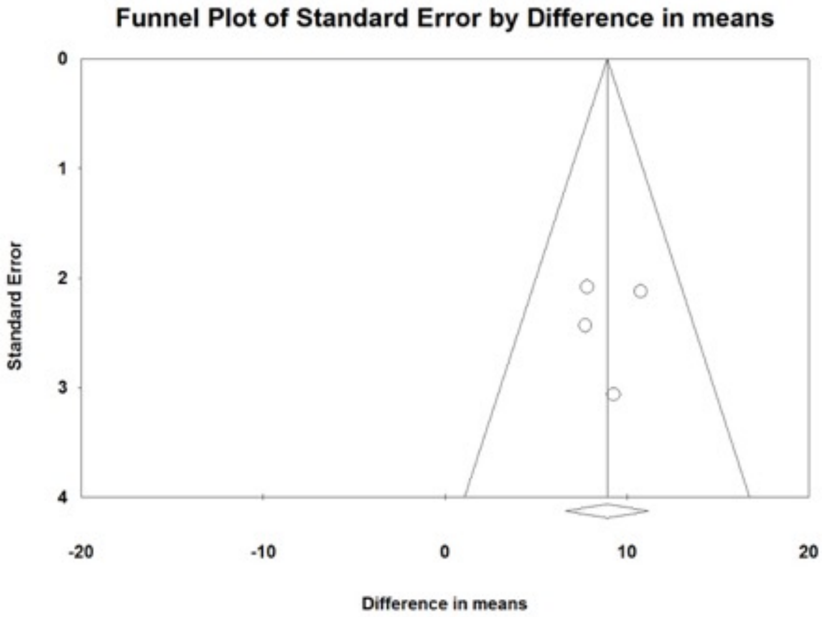

APPENDIX FIGURE 3. Changes in UA, supine position (mm^3). Enlargement of upper airway following bimaxillary advancement surgery. Funnel plot

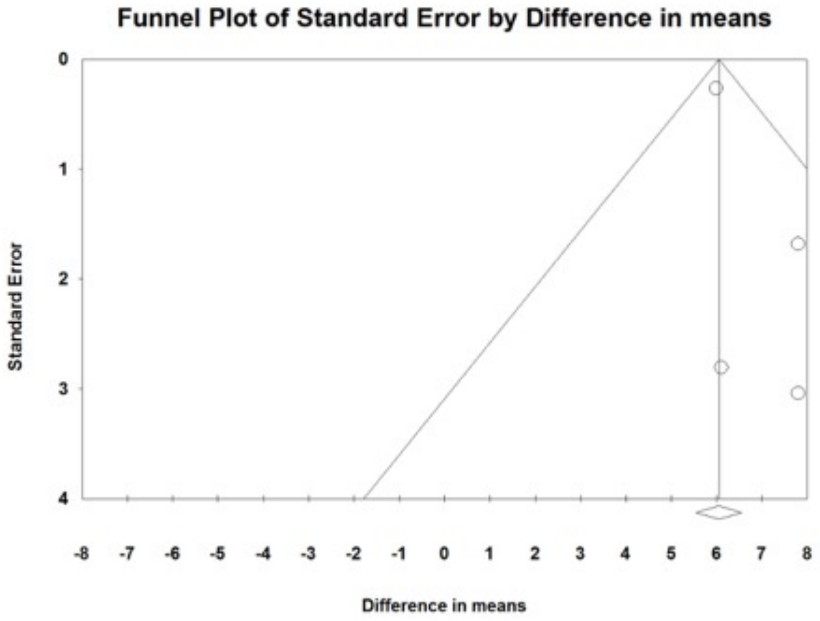

APPENDIX FIGURE 4. Changes in AHI (events/hour). Decrease in apnea/hypoapnea index following bimaxillary advancement surgery. Funnel plot

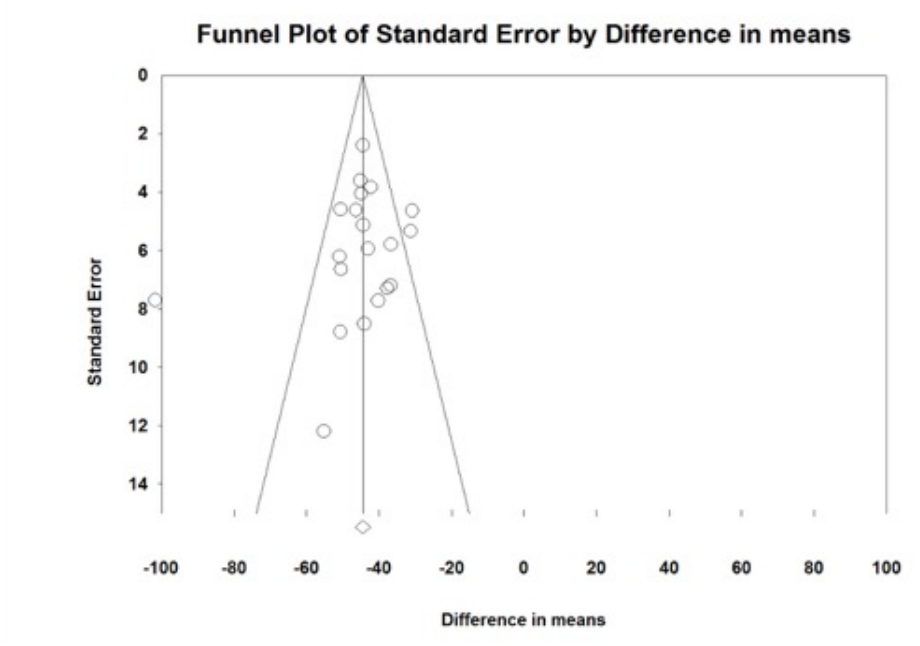

APPENDIX FIGURE 5. Changes in RDI (events/hour). Decrease in respiratory disturbance index following bimaxillary advancement surgery. Funnel plot

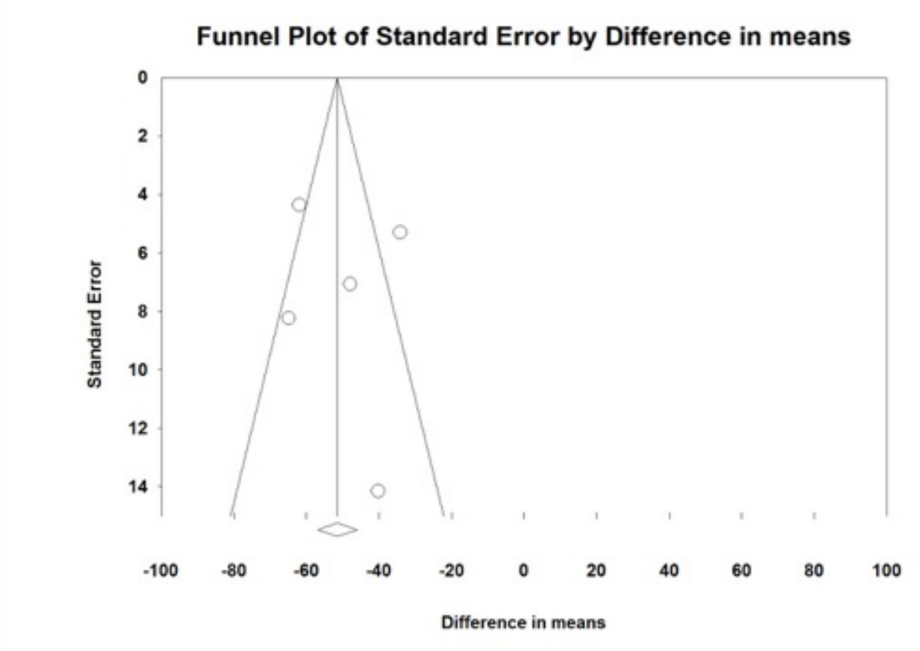

APPENDIX FIGURE 6. Changes in O2 Sat (%). Increase in oxygen saturation index following bimaxillary advancement surgery. Funnel plot

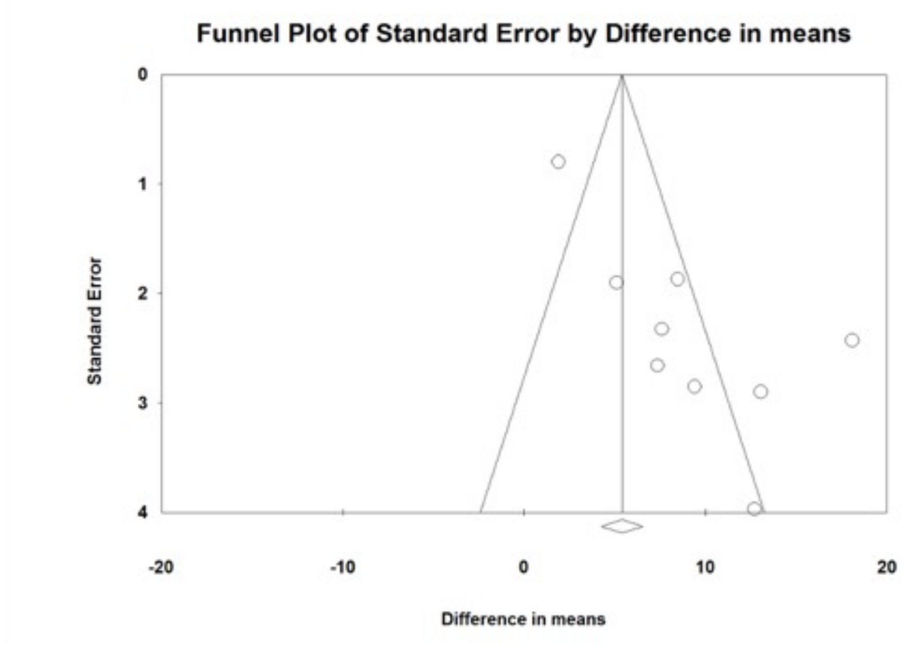

APPENDIX FIGURE 7. Changes in ESS. Decrease in Epworth sleepiness scale following bimaxillary advancement surgery. Funnel plot

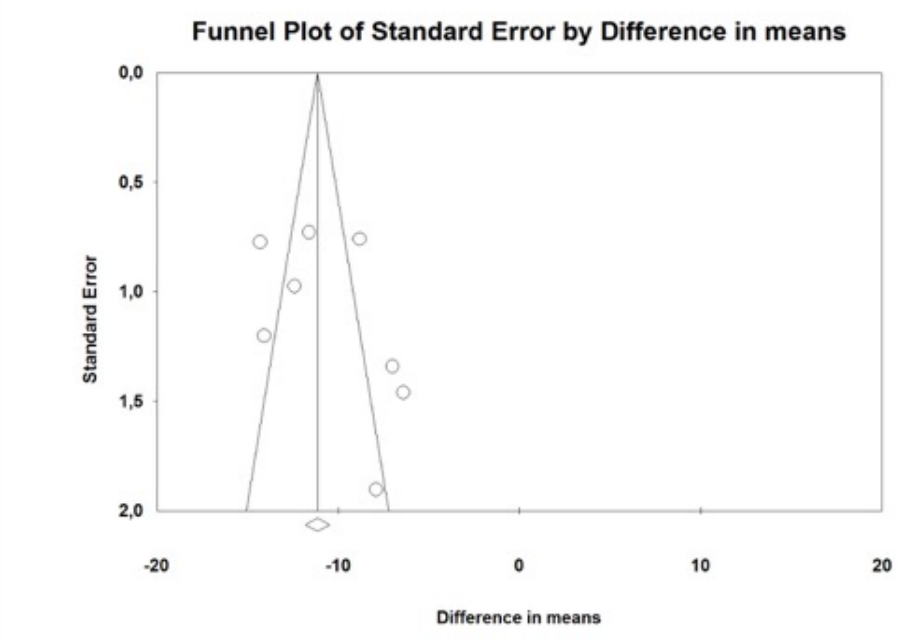

Appendix Table 1. Search strategy for identifying studies in primary electronic databases

Bimaxillary surgery AND “obstructive sleep apnea” AND (apnea OR “posterior airway space” OR airway OR “upper airway” OR "Pharyngeal space " OR PAS OR pharynx OR “hyoid bone”)
Bimaxillary surgery AND OSA AND (posterior airway space OR airway OR upper airway OR "Pharyngeal space / airway" OR PAS OR pharynx OR hyoid bone)
Retrognathia AND “obstructive sleep apnea” AND (apnea OR “posterior airway space” OR airway OR “upper airway” OR "Pharyngeal space " OR PAS OR pharynx OR “hyoid bone”)
Retrognathia AND OSA AND (posterior airway space OR airway OR upper airway OR "Pharyngeal space / airway" OR PAS OR pharynx OR hyoid bone)
“Mandibular advancement” AND “obstructive sleep apnea” AND (apnea OR “posterior airway space” OR airway OR “upper airway” OR "Pharyngeal space " OR PAS OR pharynx OR “hyoid bone”)
“Mandibular advancement” AND OSA AND (posterior airway space OR airway OR upper airway OR "Pharyngeal space / airway" OR PAS OR pharynx OR hyoid bone)
“Orthognathic surgery” AND “obstructive sleep apnea” AND (apnea OR “posterior airway space” OR airway OR “upper airway” OR "Pharyngeal space " OR PAS OR pharynx OR “hyoid bone”)
“Orthognathic surgery” AND OSA AND (posterior airway space OR airway OR upper airway OR "Pharyngeal space / airway" OR PAS OR pharynx OR hyoid bone)
"Malocclusion Angle class II" AND "obstructive sleep apnea" AND ("apnea" OR posterior airway space OR airway OR "upper airway" OR "pharyngeal space" OR PAS OR pharynx OR "hyoid bone")
“Malocclusion, Angle class II” AND OSA AND (posterior airway space OR airway OR upper airway OR "Pharyngeal space / airway" OR PAS OR pharynx OR hyoid bone)

Appendix Table 2. Excluded articles

Author/year	Exclusion criterion
**Riley et al., 1993**	Narrative review or letters to the editor,
**Yu et al., 1994**	Measures upper airway changes through teleradiography
**Turnbull et al., 2000**	Does not answer PICO question
**Sickels et al., 2002**	Sample includes patients aged under 18 years
**Eggensperger et al., 2005**	Measures upper airway changes through teleradiography
**Goncalves et al., 2006**	Measures upper airway changes through teleradiography
**Foltán et al., 2007**	Measures upper airway changes through teleradiography
**Friedman et al., 2008**	Narrative review or editorial
**Lin et al., 2008**	Does not answer PICO question
**Friedman et al., 2009**	Does not answer PICO question
**Boyd et al., 2009**	Narrative review or editorials
**Abramson et al., 2010**	Does not answer PICO question
**Aurora et al., 2010**	Does not answer PICO question
**Demetriades et al., 2010**	Measures upper airway changes through teleradiography
**Guijarro-Martínez et al., 2011**	Does not answer PICO question
**Schendel et al., 2011**	Narrative review or editorial
**Li et al., 2011**	Narrative review or editorial
**Carvalho et al., 2012**	Does not answer PICO question
**Jaspers et al., 2012**	Sample size under 10 patients
**Raunio et al., 2012**	Does not answer PICO question
**Raffaini and Pisaini et al., 2013**	Does not answer PICO question
**Valladares-Neto et al., 2013**	Does not answer PICO question
**Brunneto et al., 2014**	Does not answer PICO question
**Burkhard et al., 2014**	Does not answer PICO question
**Ubaldo et al., 2015**	Does not answer PICO question
**Torres et al., 2016**	Measures upper airway changes through teleradiography
**Riepponen et al., 2017**	Measures upper airway changes through teleradiography

Appendix Table 3: Quality of the studies on the Newcastle-Ottawa Scale

Newcastle Ottawa Scale (Cohorts)								
**Author/year [reference]**	**SELECTION (****)**				**COMPARABILITY (**)**	**OUTCOME (***)**		
	Representativeness of the exposed cohort	Selection of the non-exposed cohort	Ascertainment of exposure	Demonstration that outcome of interest was not present at start of study	Comparability of cohorts on the basis of the design or analysis **(CBCT/TC or PSG=* , CBCT/TC and PSG=**)**	Assessment of outcome	Was follow-up long enough for outcomes to occur **(6 months)**	Adequacy of follow up of cohorts **(<20%)**
**Waite et al., (1989)[23]**	*	NA	*		*	*		*
**Conradt et al., (1997)[8]**	*	NA	*		*	*	*	*
**Riley et al., (2000)[21]**	*	NA	*		*	*	*	*
**Li et al., (2002)[15]**	*	NA	*		*	*		*
**Smatt et al., (2005)[25]**	*	NA	*		*	*	*	*
**Fairburn et al., (2007)[29]**	*	NA	*		**	*	*	*
**Lye et al., (2008)[30]**	*	NA	*		**	*	*	*
**Ronchi et al., (2010)[35]**	*	NA	*	*	**	*	*	*
**Abramson et al., (2011)[31]**	*	NA	*		**	*	*	*
**Brevi et al., (2011)[10]**	*	NA	*		*	*	*	*
**Hernández-Alfaro, et al., (2011)[22]**	*	NA	*		*	*		*
**Lin, et al., (2011)[32]**	*	NA	*	*	**	*		*
**Susarla et al.; (2011)[24]**	*	NA	*		*	*	*	
**Boyd et al., (2013)[17]**	*	NA	*		*	*	*	*
**Faria et al., (2013)[7]**	*	NA	*	*	*	*	*	*
**Faria et al., (2013)[11]**	*	NA	*	*	**	*	*	*
**Giarda et al., (2013)[26]**	*	NA	*		*	*	*	*

**Ronchi et al., (2013)[27]**	*	NA	*	*	*	*	*	
**Zinser et al., (2013)[36]**	*	NA	*	*	**	*	*	*
**Bianchi et al., (2014)[16]**	*	NA	*	*	**	*	*	*
**Hsieh et al., (2014)[33]**	*	NA	*		**	*	*	*
**Schendel et al.,(2014)[38]**	*	NA	*	*	**	*		*
**Butterfield et al., (2015a)[34]**	*	NA	*		**	*	*	*
**Liu et al., (2015)[28]**	*	NA	*		*	*	*	*
**Godday et al., (2016)[13]**	*	NA	*	*	*	*	*	*

Newcastle Ottawa Scale (Case-Controls)								
**Author/year [reference]**	**SELECTION (****)**				**COMPARABILITY (**)**	**EXPOSURE (***)**		
	Case definition adequate	Representativeness of cases	Selection of controls	Definition of controls	Comparability of cases & controls	Ascertainment of exposure	Same method of ascertainment for cases & controls	Non-response rate
**Butterfield et al., (2015b)[37]**	*	*	*	*	**	*	*	*

Appendix Table 4: Characteristics of the studies included in the qualitative analysis

Author (year)<br>[reference]	TS	N (drop-outs)<br>M/F<br>Age	BMI(kg/m^2)<br>Pre-op<br>Post-op	AHI<br>Pre-op<br>Post-op	O2 sat<br>Pre-op<br>Post-op	RDI<br>Pre-op<br>Post-op	ESS<br>Pre-op<br>Post-op	Previous ops	UA<br>(mm)/%	MMA<br>(mm)	Additional ops	T0 (pre-op)<br>T1 (post-op)<br>T2 (1st follow-up)<br>T3 (2nd follow-up)
**Waite et al. (1989)**[23]	RS	23 (0)<br>21/2<br>45 (35-64)	-	-	**Desat 90**<br>203<br>10.3	63<br>15	-	UPPP (5)	-	Max→6.9<br>Mand→12.9	SP + PT<br>(23)	T0<br>T1=1w<br>T2=6w
**Conradt et al. (1997)**[8]	PS	15 (0)<br>14/1<br>44±12	28.3 ± 3.4	T0=51.4<br>(16.9)<br>T2= 5.0 (5.8)<br>T3= 8.5 (9.4)	**<90%Sa0$_2$**<br>T0= 12.6<br>(14.6)<br>T2= 0.4 (1.1)<br>T3= 2.3 (4.1)	-	-	-	-	Max → 10<br>Mand → 10	-	T0<br>T2= 6-12w<br>T3= 1-2y
**Riley et al. (2000)**[21]	RS	40 (0)<br>33/7<br>45.6±20.7	T0=31.4<br>±6.7<br>T2 = 31.0<br>±6.4<br>T3 = 32.2<br>±6.4	-	T0=67.5<br>±14.8%<br>T2= 85.6 ±<br>4.1%<br>T3 = 80.6<br>±3.9%	T0=71.2<br>±27.0<br>T2=9.3<br>±5.4<br>T3=7.6<br>±5.1	-	-	T0= 3.7±1.6<br>T1=<br>10.1±2.1<br>T3= 6.7 ±1.9<br>(-34%)	Max→7.1<br>±1.3<br>Mand→10.8<br>±2.7	GGA<br>13.2<br>±1.8mm<br>GAHM	T0<br>T1=imm<br>T2=6m<br>T3= 50.7 ±31.9m<br>(12-146m)
**Li et al. (2002)**[15]	PS	12 (0)<br>9/3<br>47.3 ± 9.8	33.5 ± 6.2<br>32.3 ± 4.1	-	74.2% ±<br>12.0%<br>86.9% ± 6.7%	75.3±26.<br>4<br>10.4±10.<br>8	-	-	6.0±2.0<br>10.4± 3.6	MMA→10.5<br>± 1.2 mm	-	T0<br>T2=6m
**Smatt et al. (2005)**[25]	RS	18 (0)<br>15/3<br>46.6±6.06	29.23±4.14	54±20.70<br>9.65±6.7 (0-<br>41)	93 ±2.8%<br>(97-85%)<br>94.9±1.9 (97-<br>91.5%)	-	-	-	-	Mand→<br>10.66 ±2.82<br>(14-6)<br>Max→ 5.24<br>±1.8 (2.5-7.5)	GP<br>GLP<br>UP	T0<br>T2=6m
**Fairburn et al. (2007)**[29]	RS	20 (0)<br>13/7<br>29-60	>55,<16<br>>54,<16	26.8-134<br>1.6-55	56-94%<br>78-98%	-	-	UPPP (10)<br>SP (12)<br>MS(1)<br>AD+AM (2)	-	Max→10<br>Mand→10	-	T0<br>T2=3-6m
**Lye et al. (2008)**[30]	RS	15 (0)<br>13/2<br>47.93 (23-67)	32.1 (22.1-<br>40.6)	69.12 (18.7-<br>112)<br>13.87 (1.7 –<br>52.9)	76.49 (45-<br>90.6)<br>84.96 (62-<br>94.6)	-	-	-	-	Max → 8.59<br>(3.14-11.04)<br>Mand → 8.96<br>(4.93-16.59)	(12)<br>GO<br>UPPP<br>SP<br>PT	T0<br>T2=6m<br>**FOSQ**<br>T0<br>T2=4-6m
**Ronchi et al. (2010)**[35]	RS	I<br>11(0)<br>9/2<br>44.36	25.42	57.9<br>7	-	-	15.2<br>0.9	-	4.4<br>11.1	MMA at least<br>10mm	GP (9)<br>SP (8)<br>LT (2)	T0<br>T1=8-10d<br>T2=4-27m
		II<br>11 (0)	27.34	50.6<br>7.6	-	-	12.5<br>0.9	-	3.7<br>10.9	-	GP (9)<br>SP (7)	

		10/1<br>45.45									Lt (2)<br>BG (2)	
**Abramson et al. (2011)[31]**	RS	11(0)<br>9/2<br>38.9 ± 12.9	28.7 ± 5.9	-	80.5%±11.4%	48.8±27.1	-	-	12.8±5.1<br>20.6±8.7	Max→9.2 ± 2.7<br>Mand→10.1 ±2.7	GTA	T0<br>T2=3-6m
**Brevi et al. (2011)[10]**	RS	33(0)<br>32/1<br>28-69	29.9(24-38)	55.6(24.2-94)<br>10.4(10-40)	-	-	15.4(0-24)<br>1.4(0-8)	-	+7.7mm	-	AG (26)<br>MxO<br>GP (5)	T0<br>T2=6m
**Hernández-Alfaro et al. (2011)[22]**	RS	10 (0)<br>-<br>-	-	-	-	-	-	-	14.53±5.09<br>23.80±8.23	-	-	T0=1d<br>T2=146d
**Lin et al. (2011)[32]**	PS	12 (0)<br>9/3<br>33±7.7	22.4±2.7<br>21.6±2.6	35.9±18<br>4.6±4.1	83±7.2<br>90.6±3.6	-	12.4±4.5<br>6±2.3	AS	Pre:<br>59±7.9mm<br>Post:<br>52.2±7.5mm<br>(-6.8±4mm)	Max → -<br>Mand → >10mm	GGT<br>MbO	T0<br>T1=3m
**Susarla et al. (2011)[24]**	RS	23 (0)<br>17/6<br>39.3 ± 12.1	27.6 ± 5.3	-	-	53.2±22.4<br>19±12	-	-	7.5 ± 2.5<br>13 ± 3	Max→9.8 ± 2<br>Mand→10.8±2.2	GTA	T0= 1-3w<br>T2=3-6m
**Boyd et al. (2013)[17]**	RS	MMA<br>37 (0)<br>-<br>44.2±9	29.8±3.8	56.3±22.6<br>11.4±9.8	74.2±13.8<br>83.6±10.5	-	-	-	-	Max → approx. 10mm<br>Mand→ >=10mm	GGA	T0<br>T1=3-6m
		MMA+UPPP<br>35 (0)<br>-<br>45.3±11	31.5±5.9	55.7±49.2<br>11.6±10.7	80.6±9.5<br>85.7±6	-	-	-	-	-	GGA<br>UPPP	
**Faria et al. (2013)[7]**	PS	19<br>14/5<br>-	-	32.1<br>10.8	-	-	-	-	8.74<br>15.95	Max→6.16<br>Mand→6	-	T0<br>T2=6m
**Faria et al. (2013)[11]**	PS	20(0)<br>15/5<br>26-60	-	30.96<br>10.28	-	-	-	-	26%RP<br>27%RL	Max→6.16<br>Mand→6	-	T0<br>T2=6m
**Giarda et al. (2013)[26]**	RS	16 (0)<br>13/3<br>49.31±8.22	-	T0=47.1±22.5<br>T1=16.1±17.5<br>T2=10.4±5.4	T0=25±7<br>T1=10±18<br>T2=19.3 ±39.3	-	T0= 12.93 ±1.69<br>T1=2.56 ±1.99<br>T2=4.12 ±2.52	SP / TP (5)<br>UPPP (1)<br>SP/T + UPPP (3)	T0=3.73±2.1 mm<br>T1=9.7±2.7<br>T2=10.3±2.3	Max → 9.1±1.3<br>Mand → 8.9±1.8	-	T0<br>T1=6m<br>T2=48.6±25.1m

**Ronchi et al. (2013)**[27]	RS	15 (0)<br>11/4<br>42.3 ± 9.5<br>(26.3-62.5)	-	58.7 ± 16<br>8.1 ± 7.8	71%<br>90%	-	-	-	5± 2.2<br>9.5±3.3	-	AG	T0<br>T2=12m
**Zinser et al. (2013)**[36]	RS	17 (0)<br>10/7<br>38.64 ± 10.75<br>(25-63)	- -	47.94 ± 15.64<br>5.64 ± 2.09	-	-	-	(11)<br>SP (3)<br>UP (8)<br>AM (3)<br>AM + AD<br>(5)	18.13±0.63<br>24.12±0.89	Mand→11.84<br>± 1.82<br>Max→4.10<br>±0.60<br>(CCW)	GP (13)<br>SP (8)<br>IT (11)	**PSG**<br>T0<br>T2=3-4m<br>**CT**<br>T0<br>T2=3-6m
**Bianchi et al. (2014)**[16]	RS	10 (0)<br>10/0<br>45(16-59)	-	56.8<br>12.3	-	-	-	-	12.9±4.0<br>20.7±3.5	Max→10<br>Mand→10	-	T0<br>T2=6m
**Hsieh et al. (2014)**[33]	PS	16(0)<br>12/4<br>33(22-48)	22 ± 3.3<br>(17-28)<br>-	35.7<br>4.8	-	-	-	-	17.1±7.2<br>23.2±8.6	-	-	T0<br>T2=6m<br>T3=12(8)m
**Schendel et al. (2014)**[38]	PS	10(0)<br>8/2<br>46.4(35-62)	28.6	42(16-68)<br>5.17	-	-	-	-	8.91<br>19.68	Max→9.4<br>Mand→9.5	GGA<br>(mean 6<br>mm)<br>GO<br>SP<br>PIT	T0<br>T2=3m
**Butterfield et al. (2015a)**[34]	RS	15(0)<br>13/2<br>42.4(19-61)	30.33 ±<br>4.18<br>30.05 ±<br>3.78	45.5±27.6<br>7.7±6	-	-	13.15<br>6.14	AM (4)<br>AD (1)<br>GP (1)<br>SP (1)<br>UPPP (3)<br>LAUP (1)<br>MMA (1)	9.72±3.6<br>17.54±7.19	Max→8.07±<br>2.6<br>Mand→10.8±<br>2.34	GP (2)	**PSG**<br>T0<br>T2=1-29m<br>**CBCT + TX** T0<br>T2= 2-49m
**Butterfield et al. (2015b)**[37]	RS	12(0)<br>16/8<br>42.75 ±13.03	30.35 ±<br>4.10<br>29.44 ±<br>3.89	47.77±26.06<br>7.57±5.97	-	-	-	AS (7)	9.68±3.81<br>17.4±7.51	Max→8.08 ±<br>2.71<br>Mand→11 ±<br>2.45	GP (2)	T0=imm pre<br>T2=3-12m
**Liu et al. (2015)**[28]	RS	16(0)<br>15/1<br>47±10.9	29.4±5.1<br>29.6±4.1	59.8±25.6<br>9.3±7.1	80.8±7.6<br>88.9±3.4	-	19.5±2.9<br>7.1±2.6	AM y AD<br>(37%)<br>UPPP (12%)<br>CPT (13%)	-	-	-	T0<br>T2= 6m
**Godday et al. (2016)**[13]	RS	13 (0)<br>11/2<br>38.6±8.4	38.8±10.9<br>37.3±8	117.9±9.2<br>16.1±26.2	-	-	12.9±5.5<br>5±4.1	UPPP (2)	-	-	-	T0<br>T2= 9.6m (>6m)

AD = adenoidectomy; **Addit. Ops** = additional operations; **AG =** advancement genioplasty; **AHI =** apnoea/hypopnoea index (episodes/hour); **AM =** amygdalectomy; **AS** = airway surgery;**BG =** bone graft; **BMI** = body mass index**; CBCT** = cone beam computed tomography; **CCW =** counterclockwise; **CPT** = combined palate and tongue surgery; **CT =** computed tomography; **d**=days; **ESS =** Epworth sleepiness scale; **FOSQ** = Functional Outcomes of Sleep Questionnaire; **GAHM** = genioglossal advancement with hyoid myotomy and suspension; **GGA =** genioglossal advancement; **GGT** = genioglossus tubercle; **GLP**= glossoplasty; **GO**= geniotomy; **GP**= genioplasty; **GTA** = genial tubercle advancement; **imm** = immediately after surgery; **imm pre** = immediately before surgery; **IT** = inferior turbinectomy; **LAUP =** laser-assisted uvulopalatoplasty; **LT** = lower turbinate reduction; **LV** = lower volume; **m** = months; **Mand** = mandible/mandibular; **Max =** maxilla/maxillary; **mm=** millimeters; **MbO** = mandibular osteotomy; **M/F** = male/female; **MMA** = maxillomandibular advancement; **MS =** mandibular setback ; **MxO** = maxillary osteotomy; **N** = sample size; **NPH =** nasopharyngoscopy; **O2 sat** = oxygen saturation index; **Post-op** = post-operative; **Pre-op** = pre-operative; **Prev. ops** = previous operations; **PS =** prospective study; **PSG =** polysomnography; **PIT** = partial inferior turbinectomy; **PT** = partial turbinectomy; **RDI** = respiratory disturbance index (episodes/hour); **RG =** retroglossal; **RL**= retrolingual; **RP**= retropalatal; **RS =** retrospective study; **SP** = septoplasty; **T** = turbinoplasty; **TS** = type of study; T = **TV** = total volume; **TX =** teleradiography; **T0** = pre-operative; **T1** = post-operative; **T2** = 1st follow-up; **T3** = 2nd follow-up; **UA** = upper airway; **UAL =** upper airway length; **UP** = uvuloplasty; **UPPP =** uvulopalatopharyngoplasty; **UV** = upper volume; **w** = week(s); **y** = years

Appendix Table 5: Anatomical limits and seated patient conditions during 3D scanning, by study included in the meta-analysis

Author (year) [reference]	3d scan	Anatomical limits	Patient conditions during scanning
**Hernández-Alfaro, et al., (2011)**[22]	CBCT	**Not described, appear to be:**<br>- **Upper:** palatal plane projection<br>- **Lower:** lowest edge of third vertebra	- Seated<br>- Head with Frankfort horizontal plane parallel to the ground, tongue in relaxed position, mandible in centric relation occlusion biting on a wax bite wafer<br>- Patient breathing peacefully<br>- Scan duration 7 seconds
**Schendel et al.,(2014)**[38]	CBCT	- **Upper:** posterior nasal spine<br>- **Lower:** hyoid bone	- Seated<br>- Natural head position
**Butterfield et al., (2015a)**[34]	CBCT	- **Upper:** posterior nasal spine<br>- **Lower**: lower end of epiglottis<br>- **Lateral**: lateral and posterior pharyngeal walls<br>- **Posterior:** posterior edge of the tongue	- Seated<br>- Natural head position<br>- Patient asked not to swallow during scanning process
**Butterfield et al., (2015b)**[37]	CBCT	- **Upper**: posterior nasal spine<br>- **Lower**: lower end of epiglottis<br>- **Anterior:** base of tongue<br>- **Posterior:** posterior pharyngeal wall<br>- **Lateral:** lateral pharyngeal walls, right and left	- Seated<br>- Natural head position<br>- Patient asked not to swallow during scanning process

Appendix Table 6: Anatomical limits and supine patient conditions during 3D scanning, by study included in the meta-analysis

Author (year) [reference]	3d scan	Anatomical limits	Patient conditions during scanning
**Bianchi et al., (2014)[16]**	CT	- **Upper:** Hard palate<br>- **Lower:** Lower edge of hyoid<br>- **Anterior:** Junction of upper adenoid tissue and nasopharynx<br>- **Posterior:** Posterior pharyngeal wall<br>- **Lateral:** Right and left lateral pharyngeal walls	- Supine<br>- Patient awake<br>- Natural head position<br>- Patients asked to hold their breath at the end of normal inhalation
**Hsieh et al., (2014)[33]**	CT	- **Upper (Velopharynx):** from posterior nasal spine to tip of uvula<br>- **Oropharynx:** from tip of uvula to tip of epiglottis<br>- **Lower (Hypopharynx):** from tip of epiglottis to vocal cord	- Supine<br>- Patient awake<br>- Patient's head with Frankfort horizontal plane (FH) perpendicular to the ground<br>- Patients asked not to swallow, to keep their mouths shut and to maintain centric occlusion bite
**Abramson et al., (2011)[31]**	CT	- **Upper:** palatal plane projection<br>- **Lower:** base of the epiglottis	- Supine<br>- Patient awake<br>- Natural head position.<br>- Patients asked not to swallow and to hold their breath at the end of normal exhalation
**Zinser et al., (2013)[36]**	CT	- **Upper (nasal cavity)**: from ethmoidal air cells to posterior nasal spine<br>- **Nasopharynx:** from posterior nasal spine to tip of uvula<br>- **Oropharynx:** from tip of uvula to tip of epiglottis<br>- **Lower (Hypopharynx):** from tip of epiglottis to 2 cm below base of epiglottis	- Supine<br>- Patient awake<br>- Patients asked to remain still, not to swallow, to place the tongue against the incisors and to hold their breath at the end of exhalation. Mandible positioned centrally and lips relaxed.
